# Rat’s response to a novelty and increased complexity of the environment resulting from the introduction of movable vs. stationary objects in the free exploration test

**DOI:** 10.1371/journal.pone.0279006

**Published:** 2022-12-20

**Authors:** Anna Chrzanowska, Klaudia Modlinska, Katarzyna Goncikowska, Wojciech Pisula

**Affiliations:** Institute of Psychology, Polish Academy of Sciences, Warsaw, Poland; Liverpool John Moores University, UNITED KINGDOM

## Abstract

Most animals, including rats, show a preference for more complex environments. This is demonstrated particularly well when complexity increases due to the addition of new elements to the environment. The aim of the study was to investigate the reaction to novelty, understood as a change in environmental properties that involve both changes in complexity and controllability. Controllability may allow for dealing with challenges of an environment of low predictability in a way that the animal’s own activity reduces the uncertainty of environmental events. In our study, the animals underwent a spontaneous exploration test in low-stress conditions. After a period of habituation to the experimental arena, additional stationary (increased complexity) and/or movable (increased complexity and controllability) tunnels were introduced, and the reaction of the rats to the novel objects was measured. The results of the study confirmed that an increase in the complexity of the environment through the addition of objects triggers a more intensive exploratory activity in rats. However, an increased spatial complexity combined with the movability of the novel objects seems to result in increased caution towards the novelty after an initial inspection of the changed objects. It suggests that the complexity of the novelty may trigger both neophilia and neophobia depending on the level of the predictability of the novel environment and that the movability of newly introduced objects is not independent of other parameters of the environment.

## 1. Introduction

Complexity is a key aspect of any environment that a given organism encounters [1]. Environmental complexity can be related to the number of elements present in the environment, the number of available sources of stimulation, or relations between the different objects in the environment [2]. According to Godfrey-Smith [3], the best definition of complexity is very simple–complexity equals lack of homogeneity; complexity equals heterogeneity.

Under natural conditions, an environment can be predictable (e.g. day-night cycle) or unpredictable (e.g., predators, weather changes, human disturbance) [4]. Managing environmental unpredictability is one of the biggest challenges animals encounter throughout their lives [5]. When an unexpected event occurs in the surroundings, an animal must respond to it quickly by engaging in appropriate behaviors, drawing on its previous experience [5]. Berlyne [6] distinguishes the following properties of stimuli present in the environment: novelty, change, complexity and ‘surprisingness’, which initiate and sustain exploratory behaviors.

In an unpredictable environment, the elements present can change, and their properties can change at any time. In a laboratory setting, we are able to control almost every aspect of the environment. Therefore, a complex environment can be changeable or unchangeable, and every aspect of emergent novelty is controlled [7]. Novelty appearing in the environment plays an important role in shaping animal behavior. The response to a novel stimulus appearing in the environment is related to the animal’s genetic background [8] and its prior experience [9]. The relationship between brain memory systems and neotic preferences involves novelty detection in which current experiences are compared with encoded information about past events [10–12]. A discrepancy between the stored data and the current event is detected as novelty and addressed with specific behaviour [13]. The reaction to novelty may elicit neophobia (avoidance) and neophilia (approach). The former protects the animal from danger, while the latter is associated with gathering information from the environment [14]. The extent of neophobia and neophilia is related to the degree of novelty generated by the stimuli present in the environment [15].

The discussion about environmental complexity may benefit from the inclusion of the term ‘affordances’, coined by J.J. Gibson in the 1970’s [16]. At an operational level, affordances may be compared to a range of ‘options’ an environment offers its inhabitants; a range of available behaviors it provides [17]. It must be borne in mind, however, that the ability to interact with an object is closely linked to an organism’s perceptual abilities and the complexity of its nervous system. Depending on the level of its biological complexity, an organism can process and search for information at different levels of sensory, logical, and content-oriented complexity ([18], p. 35). In this context, ‘information’ means any event that plays a role in the animal’s behavioral regulation by attributing meaning to an environment-derived stimulus–provided that such information can be deciphered by the animal’s sensory apparatus ([18], p. 35). Many stimuli present in the environment may be available to the animal’s perceptual apparatus, but if the animal cannot use these stimuli as behavior-regulating factors, what occurs is stimulation and not information ([18], p. 35). In order to take advantage of what an environment has to offer, an organism must have the capacity to process information provided by the environmental stimuli it receives [19].

Uncertainty is yet another aspect of environmental complexity that every organism encounters. Uncertainty is a psychological state of the organism that occurs when it is faced with events occurring in the environment, the consequences of which are difficult to predict [20]. At every level of biological complexity, organisms must manage uncertainty linked to the signals or stimuli they receive from their environments and the possible consequences of actions triggered by those signals [21]. Based on their previous experience, individuals form expectations as to future events. These expectations play a major role in the reaction to new stimuli and their interpretation [21]. The ability to manage unfamiliar and novel situations is of fundamental importance in complex environments. Some researchers believe that the need to manage environmental uncertainty has not been given sufficient consideration in the discussion on what determines animal behavior [5]. Environmental uncertainty undoubtedly involves predation risk, among other things. Predation is also the main selection factor in the evolutionary processes shaping animal morphology and behavior (e.g., [22]). The risk of falling prey to predators may also vary seasonally, and in some cases, it may change from one minute to the next, so animals must exhibit behavioral flexibility in order to adjust their predator-avoidance strategies to the ever-changing situation [22].

Studies show that rats prefer more complex environments [23, 24]. Moreover, complex environments help reduce anxiety and increase the rat’s activity [25], stimulating novelty-seeking behaviors [26]. The results of experiments suggest that rats prefer environments where the level of complexity is higher than the level they previously encountered [27, 28]. In our previous experiments, environmental complexity was manipulated by increasing the number of elements (addition of tunnels) or decreasing the number of elements (removal of tunnels) in the test environment. Increasing the number of tunnels in the test phase triggered a significant change in the animals’ behavior. The rats spent more time near the new tunnels, sniffed them and came into contact with them, climbed on top of them and hid inside them. Our results suggest that rats exhibit positive reactions to new objects which do not trigger a stress response [29–33]. An animal confronted with a new stimulus in low-stress conditions is likely to engage in an activity involving approaching the source of change and exploring the novel element(s) in the environment [18 (p. 75)].

In laboratory conditions, we are able to manipulate environmental complexity. One of our previous studies focused on the impact of affordances in an enriched living environment on the animals’ behavior in an exploration test [7]. It turned out that the rats kept in standard laboratory cages (with no enrichment) exhibited a higher level of exploratory behavior during the test than the rats kept in enriched environments. This suggests that rats previously kept in standard laboratory cages needed more time to familiarize themselves with the test environment [34]. It is also possible that depriving them of the possibility of interacting with various objects in the home cage resulted in an increased likelihood that they would explore new objects in the test arena [35–37]. Although rats kept in an enriched environment are more active and exhibit more exploratory behaviors while they stay in these kinds of conditions [e.g., 38, 39], it seems that rats who are deprived of such opportunities in their living cages compensate for their need for exploration with more heightened exploration when they are given a chance.

Studies suggest that another important feature of an animal’s environment is the possibility of controlling it. Controllability is defined as the ability of animals to alter aspects of their environment (e.g., by moving or breaking items in their surroundings)—[40]. Captive animals will use controllable items more frequently than items they cannot control [41], and control over the environment may attenuate physiological stress responses [42, 43]. Sambrook & Buchanan-Smith [44] even suggest that controllability of the environment is more important than complexity when considering environmental enrichment for captive animals. Additionally, controllability may allow for dealing with challenges of an environment of low predictability in a way that the animal’s own activity reduces the uncertainty of environmental events (cf. [5]). Wild rats (*Rattus norvegicus)* inhabit almost all land environments. They adapt easily to the surrounding conditions thanks to their biological predispositions, omnivorous diet and behavioral flexibility [45]. Their living environment is characterized by a high degree of changeability, unpredictability and, therefore, uncertainty. During exploration, rats gather information about the surrounding environment [46]. However, the exploratory behaviors they exhibit are not homogenous. Berlyne [47] introduced the term ‘diversive exploration’, which is aimed at gathering information about the surroundings, and ‘specific exploration’, which involves examining new objects by the animal. Renner and Seltzer [48] divided rats’ exploratory behaviors into general exploration and examination of novel objects. As in Berlyne [47], the term ‘exploration’ was used here in the general sense of describing a range of behaviors including moving from one place to another and sniffing, while the term ‘investigation’ (i.e. ‘specific exploration’) was used to denote interactions with specific aspects of the environment such as objects that are sources of stimulation.

Our present work is a continuation of a series of studies on the various aspects of environmental novelty in rat behavior regulation [27, 28]. This study is aimed at the analysis of the reaction to novelty, understood as a change in environmental properties that involve both change in complexity and controllability (the presence of movable and unmovable objects). Therefore, the novelty did not entail increasing or decreasing environmental complexity by adding or removing elements only but also involved providing rats with the possibility of manipulating movable objects, thereby allowing them to regulate stimulation originating in the environment.

We expected that novelty in the form of increased environmental complexity and change in the properties of the environment would have an impact on the animals’ behavior, resulting in ‘specific exploration’ [47]. Movable tunnels introduce greater complexity and controllability on the environment but also account for greater uncertainty. These factors would likely motivate rats to explore more extensively than in the case of the stationary tunnels which only introduce more complexity.

Additionally, movable objects were expected to provide animals with the possibility to control their environment by manipulating the objects. An increase in environmental complexity (adding tunnels and replacing stationary tunnels with movable ones) provides stimuli bearing the features identified by Berlyne [6]. It could therefore be assumed that this type of experimental manipulation would expose the animals to challenges posed by the need to function in a complex environment. Animals can provide themselves with sensory reinforcement different from the reinforcement they receive when interacting with stationary elements. Encountering movable objects and the resulting stimulation due to the animal’s own activity should have a rewarding value (cf. [49]).

The experiment was carried out in low-stress conditions, with the new objects being a source of positive stimulation, which allowed us to assume the rewarding value of novelty thus introduced. Low-stress conditions were secured by a long habituation phase followed by the introduction of low-intensity novelty [50]. During the experiment, the rats were free to explore the experimental arena and were allowed to enter the transporter (a safe, familiar environment—cf. [51]) at all times. The low-stress experimental conditions enable animals to express a variety of behavioral species-specific repertoire.

## 2. Materials and methods

### Animals

The sample consisted of 35 male Lister Hooded rats. The rats were bred and housed in the vivarium of the Institute of Psychology, Polish Academy of Sciences, Warsaw, Poland. At the beginning of the study, the rats were approx. 90 days old and weighed approx. 350g.

The rats were housed in groups of 3–4 in Tecniplast© Eurostandard Type IV cages (610mm×435mm×215mm) with dust-free softwood granules Tierwohl Super© as bedding. They had ad libitum access to water and standard laboratory fodder (Labofeed H, WP Morawski, Kcynia, Poland). The day/night cycle was set at 12/12h (lights-on at 8.00 a.m.). The temperature was maintained at a constant 21-23ºC, and humidity at 45–60%. Prior to the experiment, the cages were cleaned once a week. However, in order to ensure that the experimental procedure was not disturbed, the cages in which the test animals were kept were cleaned just before the start of the behavioral test and again after the test was completed. The study took place daily between 11 a.m. and 2 p.m. The rats were always tested in the same order.

All the rats were housed, bred, and taken care of in accordance with the Regulation of the Polish Minister for Agriculture and Rural Development of 14 December 2016 on laboratory animal care. The experimental procedures had been approved by the First Local Committee for Ethics in Animal Experimentation in Warsaw, Poland, permit #1115/2020.

### Procedure

The experiment followed the protocol for conducting tests on rats in low-stress conditions [50]. Low-stress conditions were ensured by prolonged habituation prior to the start of the testing phase, the introduction of low-intensity novelty, and conducting the study in the dark. The rats were allowed to enter the transporter during the entire study. The experimental apparatus was the same as the apparatus used in our previous studies [7, 27, 50]. The experimental manipulation consisted in increasing the number of tunnels or changing the properties of the tunnels, depending on the experimental group. Therefore, the introduction of novelty in the form of movable tunnels did not involve placing completely new objects in the experimental arena but rather consisted in changing the properties of elements with which the animals had been familiarized before.

**Fig 1:**
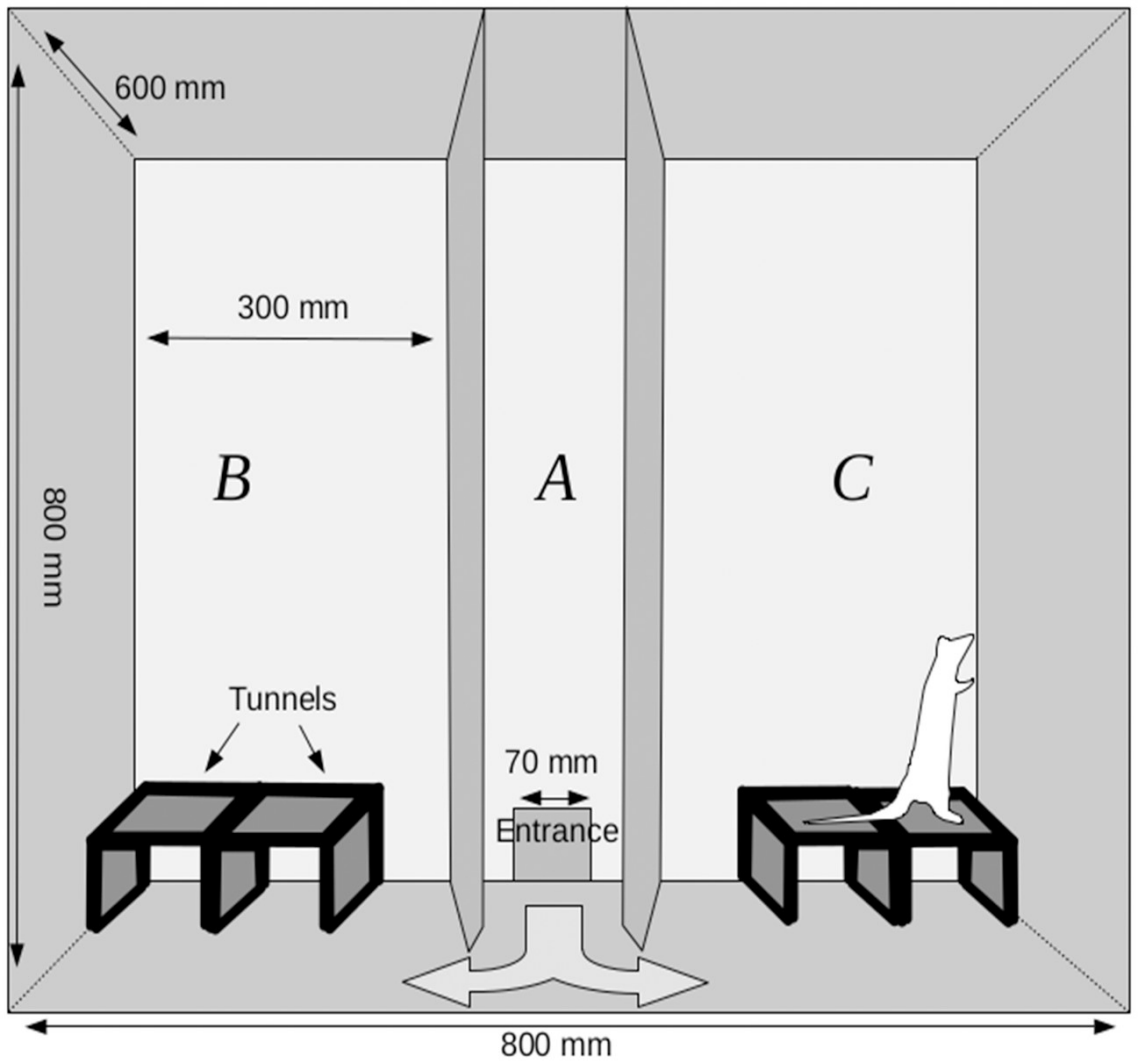
A schematic view of the experimental chamber. A transporter was set up at the entrance. The rat had free access to the transporter at all times. In Zone B the arrangement of the tunnels was the same all the time, in Zone C, on the test days (T1, T2, T3), it was changed depending on the test group.

The experimental chamber (Fig 1) was a box measuring 800mm×600mm×800mm. A detailed description has been provided elsewhere [27, 50]; therefore, we show only a general outline of the device. The chamber was divided into three zones: A, B, and C, with two walls running perpendicularly to its longer side. The partition walls between the zones had triangular openings (120mm×140mm) at the bottom, which enabled free movement between the chamber parts. There was a hole curved in the back wall of the chamber, which served as an entrance for animals going from the transporting device into the chamber. The front of the chamber was made of transparent plexiglass, and it could be lifted to obtain full access to the experimental arena. The entire chamber was covered with a layer of washable varnish. There were tunnels (200mm×120mm×80mm) placed in zones B and C made of hardwood covered with washable paint. In contrast to the most frequently used two-dimensional experimental settings, these tunnels provide a complex three-dimensional environment. The central zone (A) was left empty.

At the start of each trial, a small cylindrical cage (the ‘transporter’– 60mm in diameter with doors 120mm high and 100mm wide) with the tested animal inside was placed by the entrance to zone A. The entrance door was then lifted, and it was left open until the end of the trial. The animal was free to stay in the transporter or leave it to explore the chamber. The first seven trials were habituation trials during which the apparatus was arranged in the same way: in zones, B and C, the setting of the objects was the same (two stationary tunnels in each of the two chambers). There was no access to food or water in the testing apparatus.

**Fig 2:**
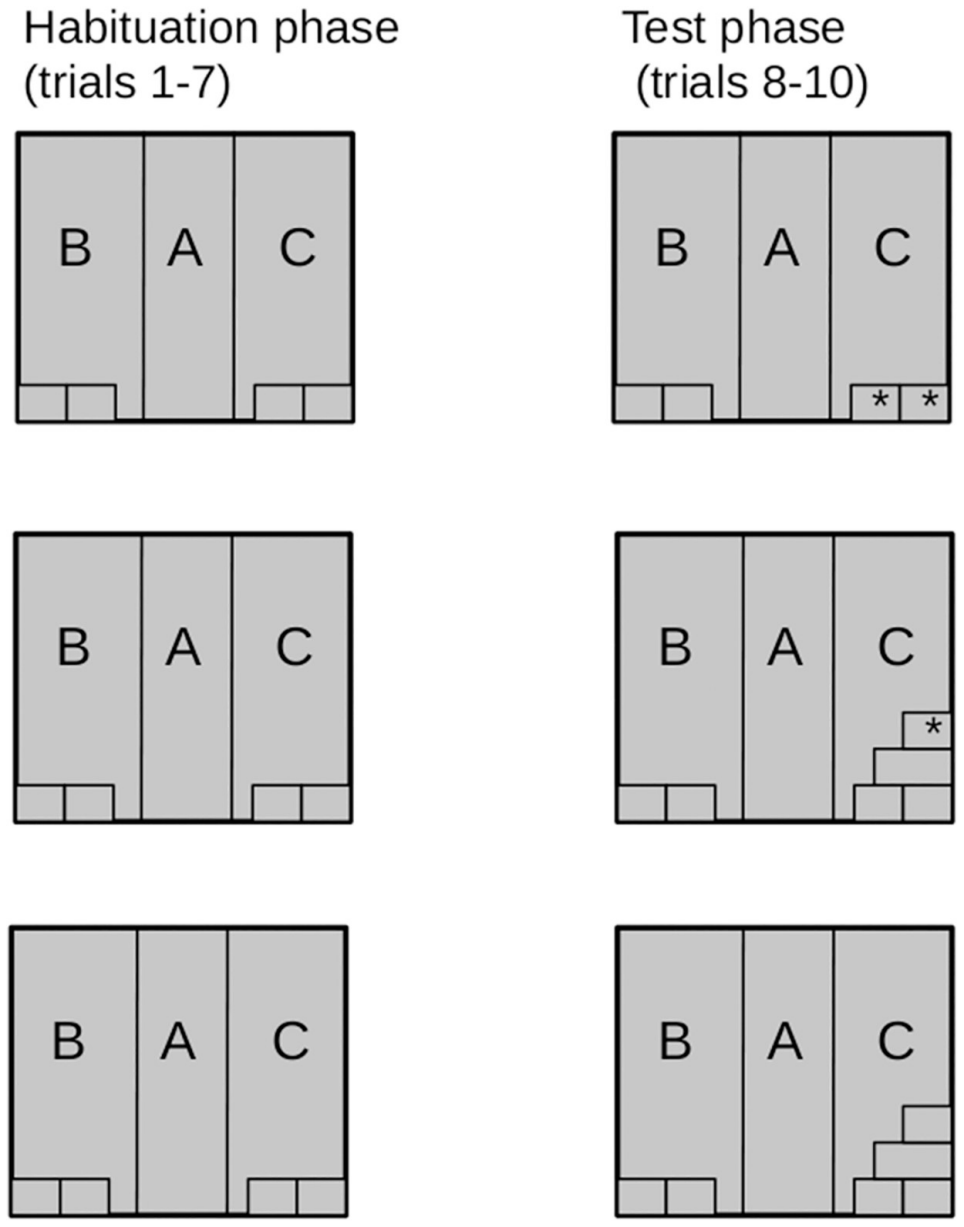
Arrangement of objects in the experimental chamber in each experimental setting. Tunnels marked with * were the see-saw-like tunnels. On the left, the setting during habituation is presented. On the right, the setup during the test days is presented: in the Mov group, two stationary tunnels were replaced by two movable tunnels (zone C). In the MovAdd group, tunnels were added, and the tunnel on the sting was movable (zone C). In the Add group, tunnels were added, and there were no movable tunnels.

The introduction of novelty took place between trials 7 and 8. The three subsequent trials were conducted with the chamber in this new arrangement (Fig 2). Each trial was 7 minutes long and was conducted for each animal once a day.

In the habituation phase, two stationary tunnels (200mm×120mm×80mm) were placed in each of zones B and C and arranged in the same manner (Fig 1). Test trials differed with regard to the configuration of the tunnels that were placed in the experimental chamber.

Setting 1 - (Mov) Movement of familiar objects in the experimental box. On the first experimental day (trial 8), movable (see-saw-like) tunnels were placed in zone C (Fig 2). These movable tunnels replaced stationary tunnels from the habituation phase. The arrangement of the tunnels in zone B remained unchanged. The Mov group consisted of 10 rats.

Setting 2 - (MovAdd) Addition of a novel movable object in the experimental box. On the first experimental day (trial 8), two supplementary tunnels were placed on top of the tunnels from the habituation phase in zone C (Fig 2). The tunnel at the very top was a movable (see-saw-like) tunnel. The arrangement of the tunnels in zone B remained unchanged. The MovAdd group consisted of 10 rats.

Setting 3 - (Add) Addition of novel stationary objects in the experimental box. On the first test day (trial 8), two tunnels placed on top of one another were added to the two tunnels in zone C. The tunnels in zone B remained in the same configuration as before. The Add group consisted of 15 rats.

To avoid the deceptive effect of lateralization or visual-auditory cues, the novelty was implemented in the right zone (as described above—zone C) for half of the test rats and in the left zone (zone B) for the remaining half (mirror image of Fig 2).

### Data processing and statistical analyses

The study was recorded in the dark with the use of a night vision camera (BSC-THC3400IR, HD-CVI 4MPX IR:30M) placed approx. 1.5m from the study apparatus. To code the behaviors on the basis of the recorded material, we used BORIS software [52], which made it possible to define selected behaviors and assess their duration and frequency. We scored the behaviors the animals engaged in during the entire experimental trial. Consequently, we were able to assign specific scores to the time of separate bouts of behaviors, their frequency, and the total time an animal spent engaging in a given behavior. The following variables were measured: (1) Time spent in the transporter (excluding the latency to leave the transporter); (2) Time spent in the unchanged zone of the chamber; (3) Time spent in the changed zone of the chamber; (4) Frequency of moving between the zones (left/right/transporter) of the chamber; (5) Time spent on contact with the tunnels in the changed zone of the chamber; (6) Frequency of contact with the tunnels in the unchanged zone of the chamber; (7) Time spent on contact with the tunnels in the unchanged zone of the chamber; and (8) Frequency of contact with the tunnels in the changed zone of the chamber.

To enhance the legibility of the results and tables, the habituation phase has been indicated as H (mean score from habituation trials 5 to 7, which served as a reference value for further analyses), while the test trials have been indicated as T1, T2, and T3, respectively. Novelty (i.e., addition of tunnels or addition and change of properties of tunnels in zone C) was introduced in the first test trial (T1).

We have decided not to present the results of the initial four habituation trials, as they serve only as the habituation phase and not as an element of a comparative analysis of the animals’ response to novelty.

The data were analyzed using a General Linear Model procedure (GLM), with repeated measurements (H, T1, T2, T3) as within-subject factors, as well group assignment (Mov, Add, MovAdd) as between-subject factors. PostHoc *t*-tests were carried out subsequently with Bonferroni correction for multiple comparisons. Differences were considered significant for *p* ≤ 0.05. Descriptive statistics of all behavioral measurements are presented in Table 1.

**Table 1:** Descriptive statistics of all behavioral measurements analyzed in this study. Group Add—addition of novel tunnels; group MovAdd—addition of movable tunnel; group Move—replacement of familiar tunnel for movable one. H—habituation phase; T1-T2—consecutive trials.

Group	Add N = 15		Mov N = 10		MovAdd N = 10	
Trials	Mean	Std dev	Mean	Std Dev	Mean	Std Dev
Time spent in the transporter						
H	62,588	18,430	52,633	12,385	43,900	20,344
T1	37,588	20,087	44,000	15,420	28,900	14,177
T2	42,059	26,541	48,200	16,491	21,500	8,923
T3	38,294	19,274	39,800	16,199	38,600	25,726
**Time spent in the unchanged zone of the chamber**						
H	125,588	19,162	138,200	18,887	137,500	42,023
T1	69,118	20,624	106,800	18,152	78,500	26,983
T2	97,118	38,498	118,700	37,547	103,900	32,285
T3	86,235	34,523	127,000	46,671	127,100	34,598
**Time spent in the changed zone of chamber**						
H	126,607	23,890	101,533	24,014	109,333	25,735
T1	217,059	36,689	137,900	28,838	222,200	48,371
T2	208,059	65,480	140,600	44,488	187,200	51,963
T3	224,824	47,870	142,700	35,214	164,600	42,380
**Frequency of moving between the chamber zones (left/right/transporter)**						
H	14,569	2,021	22,967	3,245	20,633	3,008
T1	12,824	2,580	20,100	5,131	17,900	3,213
T2	13,176	4,348	21,900	2,846	17,800	2,394
T3	13,471	3,064	22,300	4,762	19,900	3,604
**Time spent on contact with the tunnels in the changed zone of the chamber**						
H	76,059	19,084	60,333	16,555	66,333	20,292
T1	167,765	34,485	96,200	32,495	181,000	50,767
T2	160,294	56,553	92,600	40,722	144,500	48,153
T3	182,000	43,742	89,600	34,900	122,200	32,775
**Frequency of contact with the tunnels in the unchanged zone of the chamber**						
H	6,568	1,224	9,467	1,229	9,600	3,813
T1	4,882	1,933	8,500	2,321	6,700	2,584
T2	5,118	1,900	8,000	2,625	7,600	1,647
T3	4,706	1,490	8,500	1,080	8,400	2,119
**Time spent on contact with the tunnels in the unchanged zone of the chamber**						
H	78,098	16,652	79,333	11,389	82,567	29,019
T1	47,706	25,544	69,000	15,195	41,600	15,778
T2	53,118	18,927	74,100	23,965	68,100	27,201
T3	51,235	23,212	80,800	31,094	79,400	27,925
**Frequency of contact with the tunnels in the changed zone of the chamber**						
H	6,235	1,678	7,900	1,899	7,900	2,091
T1	7,529	1,972	9,900	2,079	10,800	2,616
T2	6,647	2,499	9,200	1,874	9,300	1,767
T3	7,235	2,251	8,300	4,191	8,700	1,703

## 3. Results

### Time spent in the transporter

The analysis showed the main effect of the trial: *F*(3,96) = 5.165, *p* = 0.002, Eta^2^ = 0.139 (Wilks’ Lambda) and a main effect of group: *F*(2,32) = 6.262, *p* = 0.005, Eta^2^ = 0.281. There was no interaction effect of trial and group.

A post hoc analysis showed a significant **decrease** in the time spent in the transporter **in the first test trial** compared to the habituation phase (*t* = 3.058; *p* = 0.017, Cohen’s *d* = 0.517), a significant **decrease** in time spent in the transporter **in the second test trial** compared to the habituation phase (*t* = 2.913; *p* = 0.004, Cohen’s *d* = 0.598) and a significant decrease **in the third test trial** compared to the habituation test (*t* = 2.913; *p* = 0.027, Cohen’s *d* = 0.492). There were no changes in the time spent in the transporter between the test trials.

There were also differences in the time spent in the transporter between the groups. Rats from the Add group spent more time in the transporter compared to the rats from the MovAdd (*t* = 3.491; *p* = 0.004, Cohen’s *d* = 0.590). There were no differences between the group Mov and Add group (*p* = 0.188) and group Mov and group MovAdd (p = 0.490).

### Time spent in the unchanged zone of the chamber

The analysis showed a significant trial by group interaction: *F*(3, 96) = 2.804, *p*<0.015, Eta^2^ = 0.149 (Wilks’ Lambda) and the main effect of the trial: *F*(3, 96) = 12.722, *p*<0.001, Eta^2^ = 0.0.284 (Wilks’ Lambda).

For the MovAdd rats, a post hoc analysis showed a significant **decrease** in the time spent in the unchanged zone of the chamber **in the first trial** compared to the habituation phase (*t* = 5.283; *p*<0.001, Cohen’s *d* = 1.670) and a significant decrease **in the third trial** compared to the first trail (*t* = 4.352; *p*<0.002, Cohen’s *d* = 1.566). No differences were observed between the trials in the Mov (H-T1, *p* = 0.395; T1-T2, *p* = 1.000; T2-T3, *p* = 1.000) and Add groups (H-T1, *p* = 1.000; T1-T2, *p* = 1.000; T2-T3, *p* = 1.000).

### Time spent in the changed zone of the chamber

**Fig 3:**
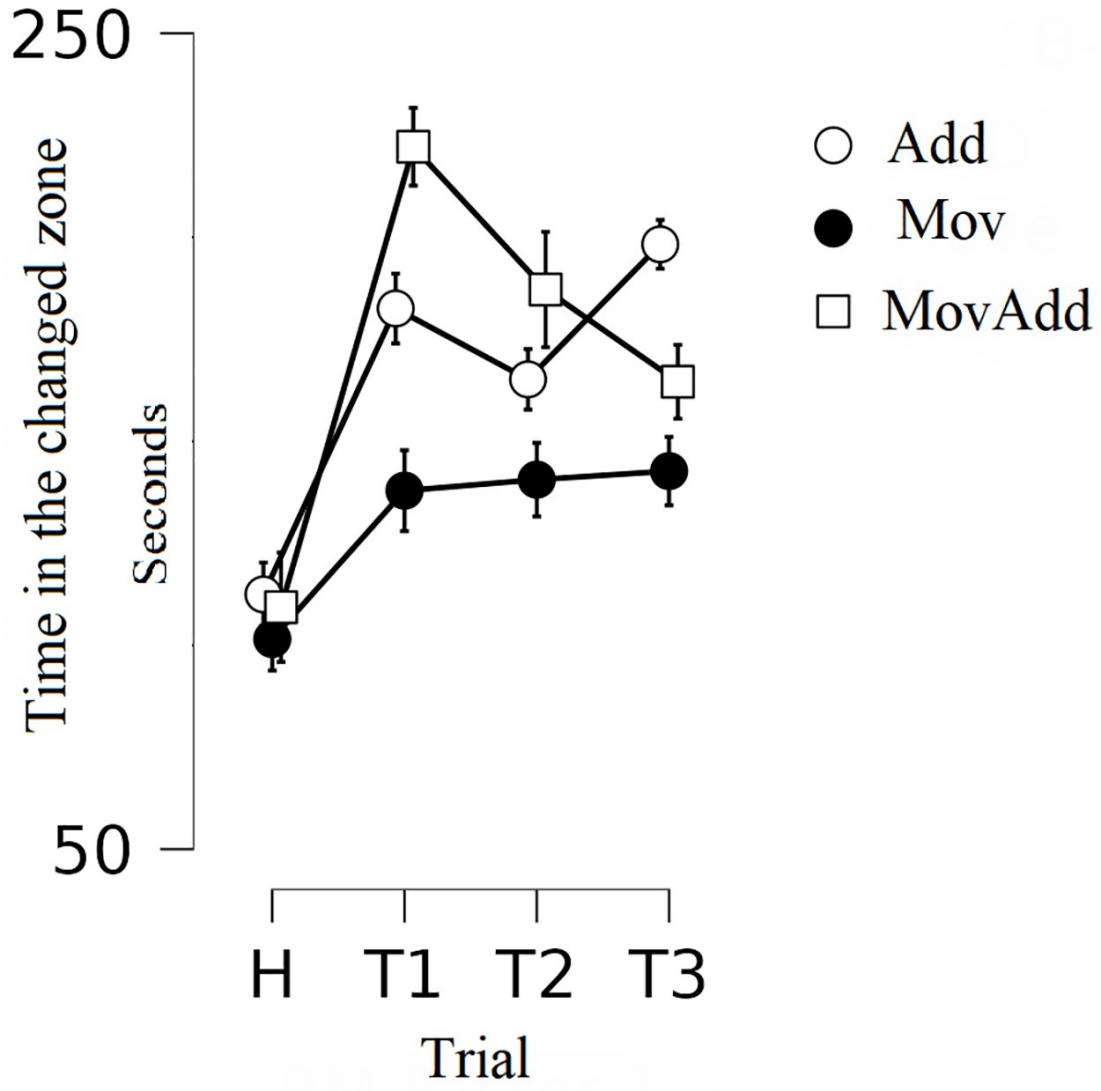
Mean time spent by rats in the changed zone of the chamber. Group Add: addition of novel stationary objects in the experimental box, group Mov: movement of familiar objects in the experimental box, group MovAdd: addition of a novel movable object in the experimental box. Error bars represent standard error (SE).

The analysis showed a significant trial by group interaction: *F*(3, 96) = 4.757, *p*<0.001, Eta^2^ = 0.229 (Wilks’ Lambda) and the main effect of trial: *F*(3, 96) = 36.563, *p*<0.001, Eta^2^ = 0.533 (Wilks’ Lambda)–Fig 3.

For the Add rats, a post hoc analysis showed a significant **increase** in the time spent in the changed zone of the chamber **in the first test trial** compared to the habituation phase (*t* = 6.146; *p*<0.001, Cohen’s *d* = 1.701), a significant **increase in the second test trial** compared to the habituation phase (*t* = 4.623; *p* = 0.001, Cohen’s *d* = 1.399) and a significant **increase in the third test trial** compared to the habituation phase (*t* = 7.526; *p* = 0.001, Cohen’s *d* = 2.239). There were no differences between the test trials.

For the MovAdd rats, a post hoc analysis showed a significant **increase** in the time spent in the changed zone of the chamber **in the first test trial** compared to the habituation phase (*t* = 8.028; *p*<0.001, Cohen’s *d* = 2.913), a significant **increase in the second test trial** compared to the habituation phase (*t* = 5.576; *p* = 0.001, Cohen’s *d* = 1.899) and a significant **increase in the third test trial** compared to the habituation phase (*t* = 3.957; *p* = 0.010, Cohen’s *d* = 1.576). There is also a significant **decrease** in the third test trial compared to the first test trial (*t* = 4.124; *p* = 0.005, Cohen’s *d* = 1.266). No differences were observed in the Mov group (H-T1, *p* = 0.705; T1-T2, *p* = 1.000; T2-T3, *p* = 1.000).

### Frequency of moving between the chamber zones (left/right/transporter)

The analysis showed a significant main effect of trial: *F*(3,96) = 5.533; *p* = 0.002; Eta^2^ = 0.147 (Wilks’ Lambda) and a main effect of group: *F*(2,32) = 3.603, *p*<0.039, Eta^2^ = 0.184, but no interactive effect of trial by the group.

Post hoc analyses showed a significant **decrease** in the frequency of moving between the zones **in the first test** trial compared to the habituation phase (*t* = 3.975; *p* = 0.001, Cohen’s *d* = 0.672).

There was also a difference in the frequency of moving between the chamber zones between the groups. Rats from the MovAdd group (*t* = 0.276; *p* = 0.047, Cohen’s *d* = 0.047) showed a higher frequency of moving between the chamber zones than rats from the Add group.

### Time spent on contact with the tunnels in the changed zone of the chamber

**Fig 4:**
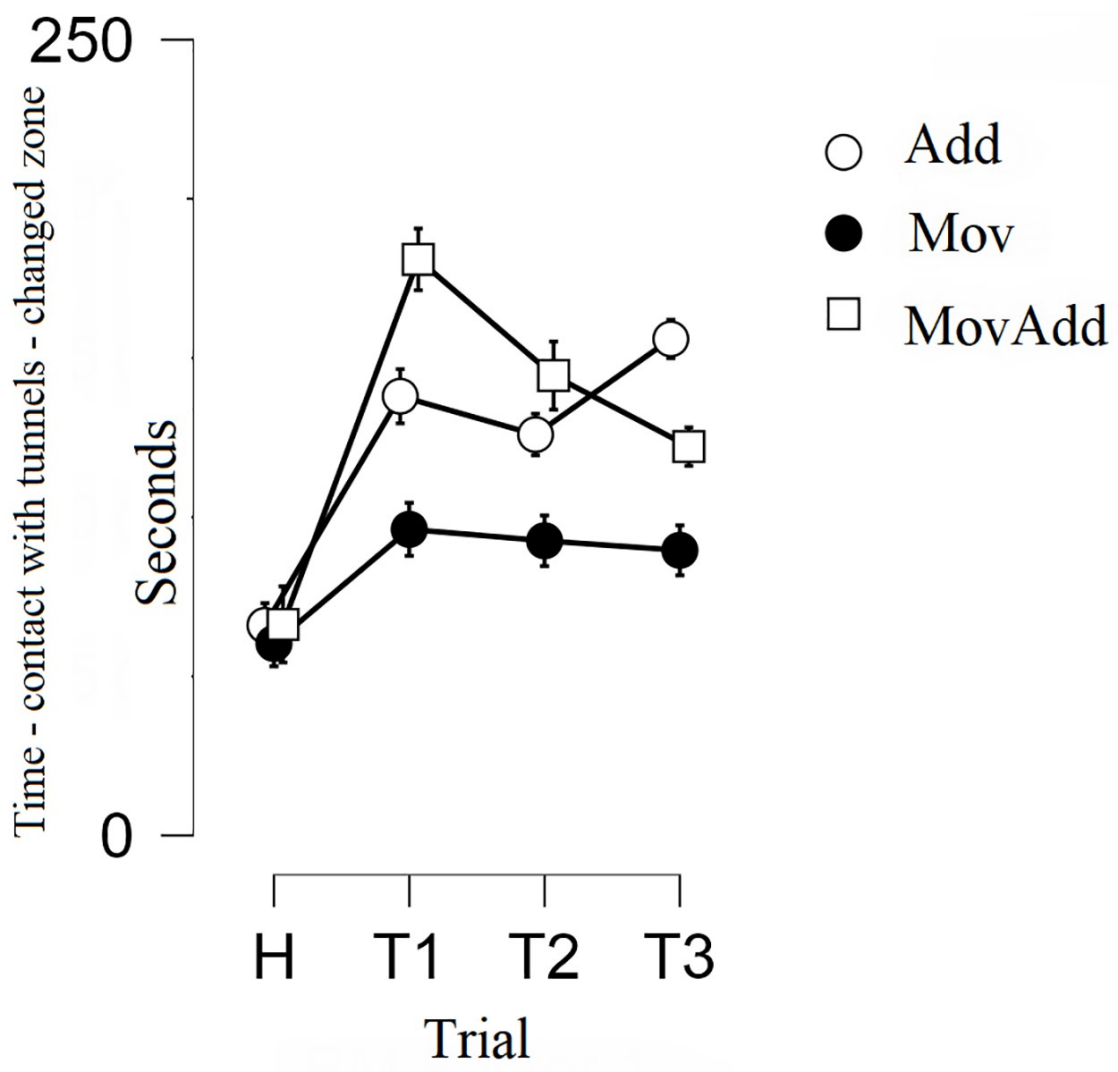
Mean time spent on the contact with tunnels in the changed zone of the chamber. Group Add: addition of novel stationary objects in the experimental box, group Mov: movement of familiar objects in the experimental box, group MovAdd: addition of a novel movable object in the experimental box. Error bars represent standard error (SE).

The analysis showed a significant trial by group interaction: *F*(3, 96) = 6.663, *p*<0.001, Eta^2^ = 0.294 (Wilks’ Lambda) and the main effect of trial: *F*(3, 96) = 46.110, *p*<0.001, Eta^2^ = 0.590 (Wilks’ Lambda)—Fig 4.

For the Add group, a post hoc analysis showed a significant **increase** in the time spent on contact with the tunnels in the changed zone of the chamber **in the first test trial** compared to the habituation phase (*t* = 7.085; *p*<0.001, Cohen’s *d* = 1.995), a significant **increase in the second test trial** compared to the habituation phase (*t* = 5.899; *p* = 0.001, Cohen’s *d* = 2.278) and a significant **increase in the third test trial** compared to the habituation phase (*t* = 8.856; *p* = 0.001, Cohen’s *d* = 2.501). No differences were observed between the test trials.

For the MovAdd group, a post hoc analysis showed a significant **increase** in the time spent on contact with tunnels in the changed zone of the chamber **in the first test trial** compared to the habituation phase (*t* = 9.218; *p*<0.001, Cohen’s *d* = 2.966), a significant **increase in the second test trial** compared to the habituation phase (*t* = 6.284; *p* = 0.001, Cohen’s *d* = 2.115) and a significant **increase in the third test trial** compared to the habituation phase (*t* = 4.491; *p* = 0.010, Cohen’s *d* = 2.050). There was also a significant **decrease** in the third test trial compared to the first test trial (*t* = 4.727; *p* = 0.005, Cohen’s *d* = 1.376).

No differences were observed in the Mov group.

### Frequency of contact with the tunnels in the unchanged zone of the chamber

The analysis showed a significant main effect of trial: *F*(3,96) = 5.412; *p* = 0.002; Eta^2^ = 0.145 (Wilks’ Lambda), and a main effect of group: *F*(2,32) = 7.230, *p*<0.003, Eta^2^ = 0.311. There was no interaction effect of trial and group.

Post hoc analyses showed a significant **decrease** in the frequency of moving between the zones **in the first test** trial compared to the habituation phase (*t* = 3.743; *p* = 0.002, Cohen’s *d* = 0.633), a significant **decrease** in the second test trial compared to the habituation phase (*t* = 2.887; *p* = 0.029, Cohen’s *d* = 0.488) and a significant **decrease** in the frequency of moving between the zones **in the third trial** compared to the habituation phase (*t* = 2.963; *p* = 0.023, Cohen’s *d* = 0.501).

There was also a difference in the frequency of contact with tunnels in the unchanged zone between the groups. Rats from the Move group (*t* = 3.569; *p* = 0.003, Cohen’s *d* = 0.603) and from the MovAdd group (*t* = 2.631; *p* = 0.039, Cohen’s *d* = 0.445) showed a higher frequency of contact with tunnels than rats from Add group.

### Time spent on contact with the tunnels in the unchanged zone of the chamber

**Fig 5:**
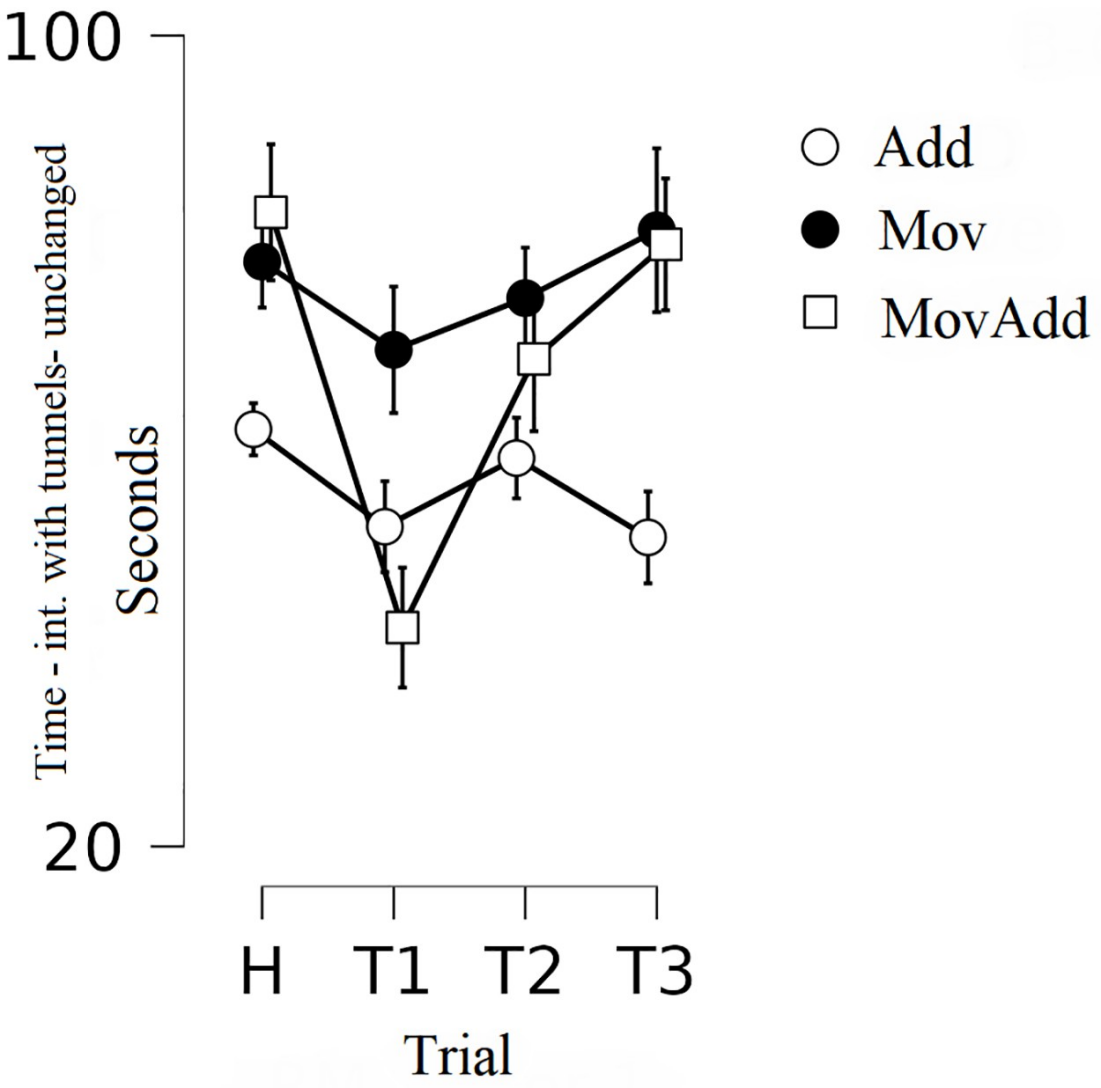
Mean time spent on contact with the tunnels in the unchanged zone of the chamber. Group Add: addition of novel stationary objects in the experimental box, group Mov: movement of familiar objects in the experimental box, group MovAdd: addition of a novel movable object in the experimental box. Error bars represent standard error (SE).

The analysis showed a significant trial by group interaction: *F*(3, 96) = 3.014, *p*<0.010, Eta^2^ = 0.159 (Wilks’ Lambda) and the main effect of trial: *F*(3, 96) = 7.562, *p*<0.001, Eta^2^ = 0.191 (Wilks’ Lambda)—Fig 5.

In the Add and Mov groups, the analyses did not show significant differences in the time spent on contact with the tunnels in the unchanged zone. In the MovAdd group, on the other hand, a post hoc analysis showed a significant **increase** in the time spent on contact with tunnels in the unchanged zone of the chamber **in the first test trial** compared to the habituation phase (*t* = 5.039; *p*<0.001, Cohen’s *d* = 1.753) and a significant **decrease** in the third test trial compared to the first test trial (*t* = 4.649 *p* = 0.001, Cohen’s *d* = 1.666).

### Frequency of contact with the tunnels in the changed zone of the chamber

The analysis showed a significant main effect of trial: *F*(3,96) = 8.979; *p* = 0.001; Eta^2^ = 0.219 (Wilks’ Lambda), but no interactive effect of trial by the group.

A post hoc analysis showed a significant **increase** in the frequency of contact with the tunnels in the changed zone of the chamber **in the first test trial** compared to the habituation phase (*t* = 4.989; *p*<0.001, Cohen’s *d* = 0.843) and a significant **increase in the second test trial** compared to the habituation phase (*t* = 3.594; *p* = 0.003, Cohen’s *d* = 0.608).

#### Effect size analysis

In addition to the RM ANOVA analyzes, we conducted an analysis of the size of the obtained effects [27]. We compared all the possible statistical effects according to the obtained Eta partial values using the Kruskal—Wallis ANOVA. The three possible statistical effects (Trial, Group, Trial x Group) were the criteria for grouping, and the number of dependent variables (behavioral measures) was the equivalent of cases.

The results obtained using the Kruskal—Wallis ANOVA (H = 5.15, *df* = 2, *p* = 0.07) showed no differences in the significance of the experimental factors for the variance of the dependent variables (behaviors measured). The lack of a significant result of this analysis indicates that the combination of novelty with object movability produced no additive effect, proving that these environmental properties do not work independently. Eta^2^ results for the significance of effects are shown in Table 2.

**Table 2:** The ranking list of statistically significant effects based on the partial Eta^2^ values. The Eta^2^ values of statistically non-significant effects have been set to “0”.

Dependent	Effect	Eta^2^
Time spent on contact with the tunnels in the changed zone of the chamber	Trial	0.590
Time spent in the changed zone of the chamber	Trial	0.533
Time spent in the unchanged zone of the chamber	Group	0.328
Time spent on contact with the tunnels in the changed zone of the chamber	Group	0.324
Frequency of contact with the tunnels in the unchanged zone of the chamber	Group	0.311
Time spent on contact with the tunnels in the changed zone of the chamber	Trial x Group	0.294
Time spent in the unchanged zone of the chamber	Trial	0.284
Time spent in the transporter	Group	0.281
Time spent in the changed zone of the chamber	Group	0.251
Time spent on contact with the tunnels in the unchanged zone of the chamber	Group	0.243
Time spent in the changed zone of the chamber	Trial x Group	0.229
Frequency of contact with the tunnels in the changed zone of the chamber	Trial	0.219
Time spent on contact with the tunnels in the unchanged zone of the chamber	Trial	0.191
Frequency of moving between the chamber zones (left/right/transporter)	Group	0.184
Time spent on contact with the tunnels in the unchanged zone of the chamber	Trial x Group	0.159
Time spent in the unchanged zone of the chamber	Trial x Group	0.149
Frequency of moving between the chamber zones (left/right/transporter)	Trial	0.147
Frequency of contact with the tunnels in the unchanged zone of the chamber	Trial	0.145
Time spent in the transporter	Trial	0.139
Time spent in the transporter	Trial x Group	0
Frequency of moving between the chamber zones (left/right/transporter)	Trial x Group	0
Frequency of contact with the tunnels in the unchanged zone of the chamber	Trial x Group	0
Frequency of contact with the tunnels in the changed zone of the chamber	Trial x Group	0
Frequency of contact with the tunnels in the changed zone of the chamber	Group	0

#### Summary of the results

*Mov group*. In the case of the Mov group, two stationary tunnels were replaced with two movable tunnels in the test phase (T1-T3). The number of tunnels did not change. No differences were observed in the amount of time spent in the changed zone or in the amount of time spent on interaction with the changed tunnels.

*MovAdd group*. In the case of the MovAdd group, the number of tunnels was increased in the test phase (T1-T3), and the tunnel placed on top of the other tunnels was a movable one. In this group, differences were observed in the amount of time spent in the changed zone of the chamber. In all test trials (T1-T3), the rats spent more time in the changed zone than they had done during the habituation phase (H). On the second test day (T2), the rats spent more time in the changed zone than on the third test day (T3). Throughout the test phase (T1-T3), the rats spent more time on contact with the tunnels than during the habituation phase (H). On the first test day (T1), they spent more time on contact with the tunnels than on the third day (T3).

*Add group*. For this group, the number of tunnels was increased in the test phase (T1-T3), but no movable tunnels were added. Throughout the test phase (T1-T3), the rats spent more time in the changed zone of the chamber than they had done during the habituation phase (H). Throughout the test phase (T1-T3), the rats spent more time on contact with the tunnels than during the habituation phase (H).

*Differences between groups*. During the test phase, the Add rats spent more time in the transporter compared to their MovAdd counterparts. Rats from Mov and MovAdd groups interacted with the tunnels in the unchanged zone of the chamber more frequently than rats from Add group. There was also a difference in the frequency of moving between the chamber zones between the groups: MovAdd rats moved between the chamber zones more frequently than the rats from the Add group.

## 4. Discussion

The purpose of our experiment was to examine the impact of novelty on animal behavior where novelty involved increasing the complexity of the environment by adding elements and/or changing the properties of elements present in the environment. In this context, novelty is ‘a change in stimulus conditions from previous experience’ [53] (p. 189). It should be emphasized that the affordances in the form of tunnels that we used in our experiment respond to the needs of the species studied (rats), as they allow rats to engage in species-typical behaviors such as climbing on top of and hiding inside the tunnels. As in our previous studies [27], an account was taken of the ecological psychology dimension.

The animals’ behavior differed between the groups, depending on the type of experimental manipulation used. In the Mov rats, no change in behavior was observed after the introduction of novelty. In this group, the manipulation involved changing the properties of the objects without increasing their number or arrangement between the habituation and test phase. On their first encounter with novelty, animals from this group could confront the change after a certain delay. The change (movability of the tunnels) was only discovered when the animals climbed the tunnel and made it move with their own body weight. It is likely that after seven days of habituation, the rats from the Mov group, due to the lack of spatial cues of the novelty, were slower to discover the change on the first test day as compared to the MovAdd group. The lack of distant cues resulted in the necessity of getting into direct proximity to the tunnel to reveal its new properties. They had to climb the tunnel and set it in motion. It may be assumed that before discovering the movability of the tunnel, the initial rats’ expectations related to the tunnel in question would be the same as in the last habituation session [21]. In our study, these expectations were formed in the seven trial/days, long habituation phase, when the rats spent a 7-minute period in a stable test arena each day. They were subsequently exposed to unexpected changes (introduction of movable elements instead of stationary ones). If the change is not immediately conspicuous (new feature–movability–of otherwise unfamiliar tunnels), it may happen that it fails to elicit an immediate response since the ability to recognize it is hindered by previously formed expectations (cf. [54, 55]).

Rats from the Add and MovAdd groups spent significantly more time in the changed zone of the chamber after the introduction of novelty. The introduction of novelty changed the animals’ behavior in such a way that the rats spent much time near the objects, coming in contact with them, sniffing or climbing them; in this case, we could observe ‘specific exploration’, to use Berlyne’s term [47]. The results we obtained may suggest, in line with what some researchers claim [56, 57], that novelty may have had a rewarding value. They also seem to confirm that under the conditions of low-stress experimental manipulation, animals are likely to investigate changes occurring in their environment ([18], p. 75) rather than exhibit a neophobic response [15]. The question remains as to why the addition of a new element in the MovAdd group resulted in a change of behavior, while no such change was observed in the Mov group described above. As demonstrated in one of our previous studies [27], rats engage in object-directed exploratory behavior where the complexity of the environment increases, i.e., when the number of tunnels goes up. The discrepancy between the expectations formed during the habituation phase and the changed environment encountered in the test phase results in a higher level of motivation to explore the new objects (cf. [21]). This allowed the MovAdd rats to detect the movability of the tunnels sooner than was the case in the Mov group, where the configuration of the tunnels did not change.

As regards the amount of time spent in the unchanged chamber and contact with the tunnels in that zone, no differences were observed in the Mov and Add groups between the habituation sessions and the test phase. However, the change was observed in the MovAdd group. Rats from that group increased the time spent in the unchanged zone and time on contact with tunnels in this zone after the introduction of novelty but decreased the time on the third test day. It seems that novelty combining move and addition of objects leads to a change of behaviour, whereas the move and addition provided separately do not have that effect. In the MovAdd group, the novelty the animals encountered had a higher degree of complexity but also a greater level of uncertainty than in the other groups. These features may have strengthened the rats’ motivation to explore, resulting in less time being also spent in the familiar, unchanged zone of the chamber [25, 27, 28].

There is also a difference in the frequency of moving between the chamber zones between groups: rats from MovAdd group moved between the chamber zones more frequently than their Add counterparts. The differences between those two groups may be due to the fact that the movable element in the MovAdd group caused a certain degree of arousal and, therefore, elicited a need to reduce uncertainty by exploring different zones of the chamber.

A decrease in the amount of time spent in the transporter after the introduction of novelty was observed in all groups, which may suggest that the change did not invoke fear in the rats. However, the Add group rats spent more time in the transporter than their MovAdd counterparts in the test phase. It may be that the MovAdd rats, when faced with novelty characterized by a higher degree of complexity and uncertainty (increase in the number of tunnels *and* addition of a movable tunnel), devoted more time to exploring the tunnels. The Add rats (increase in the number of tunnels, no new and/or movable elements) quickly familiarized themselves with the new setting and did not devote their resources to exploring the environment.

In the MovAdd group, changes were observed in the amount of time spent in the changed zone of the chamber: rats from this group spent more time in that zone and on contact with the tunnels on each test day than during the habituation phase. On the third test day, however, they spent less time there than on the first test day. This could be explained by the fact that there were several things at play simultaneously: novelty in the form of a movable element motivated the animals to engage in exploration, but it could also constitute a source of uncertainty. Changes in a complex environment are often caused by factors unknown to the animal, and the associated uncertainty emerges when an organism encounters changes whose consequences it cannot predict. There is no certainty as to whether or not a particular event will occur again in the future or when. To a certain extent, uncertainty may increase the animal’s motivation to examine the changes as they occur [38, 58]. On the first test day, curiosity and the need to explore the environment prevailed over potential fear induced by the movable object, which may explain why the rats spent a lot of time in the changed zone of the chamber and on contact with the tunnels. Our procedure secures the low-stress or no-stress conditions, effectively reduces the neophobic responses. A series of previous studies have shown that the neophobic reactions are absent after a long familiarization to the experimental arena used in our protocol [50]. However, the rats’ activity on the third test day decreased compared to the first test day. It appears that after some time, the movable element may have reduced the attractiveness of the environment which may be link to other factors than novelty of the objects, e.g., specific reaction to the movability. This phenomenon may be interpreted in terms of predation risk assessment [22], which in this case may have made interaction with the movable object more threatening than rewarding. Rodents and other small mammals can be prey for a variety of predators, including birds or other mammals. Thus, they must adapt to varying spatial predation risks [59]. Faced with a predatory threat, rodents, including rats, exhibit various behaviors such as flight, avoidance or freezing–species-specific defense response [60]. Confrontation with new objects or places is of key importance for the animal’s survival–a novelty in the environment may provide new opportunities, but, equally, it may pose a threat [45].

The results obtained for the Mov group show no difference in the amount of time spent in the changed zone of the chamber and on contact with the tunnels between the habituation phase and the test phase. It seems, therefore, that the introduction of the movability of an element does not, in itself, result in a greater rewarding value of the changed tunnels, which renders this type of manipulation very different from changes involving spatial rearrangement or an increase in the complexity of the test environment. However, we would point out that the addition of movability and spatial rearrangement occurring at the same time (as in the MovAdd group) translated into changes in the behavioral characteristics observed. Our results demonstrate that environmental change involving the introduction of movability of the manipulated objects is not independent of, but rather interacts with, other parameters of the environment, such as its complexity and spatial arrangement. This conclusion is supported by the results of the analysis of the effect sizes, which show that the movability of the tunnels does not augment the positive effect of the increase in environmental complexity provided by the addition of tunnels but rather interacts with it in a complex multidirectional way.

Based on the results of our study, it can be concluded that the increase in environmental complexity contributes to animal welfare. Rats have the opportunity to carry out species-typical behaviors, which is manifested by an increase in exploratory behavior. However, it can be assumed that the introduction of movable elements can lead to increased environmental unpredictability and may contribute to elevated stress levels, at least at the initial stage of the familiarization with novel objects.

## 5. Conclusions

The results of our study show that the characteristics of the novelty introduced to the environment play a role in behavioral regulation in rats. The experimental manipulation we used involved different types of novelty: an increase in the test environment’s complexity (addition of objects), a change in the properties of familiarized elements and a combination of these two scenarios.

The changes made to the test environment were ‘affordance-inviting’, to use a term coined by Withagen and colleagues [61]. The results obtained for the Add and MovAdd groups seem to corroborate this assumption. The results of our previous studies [27, 28] are also confirmed, which showed that an increase in the complexity of the environment through the addition of tunnels triggers a more intensive exploratory activity in rats.

Our results indicate that the introduction of movability (as a new property of a familiar object) does not, as such, lead to a greater rewarding value being attributed to the changed tunnels. However, combining the introduction of movability with spatial rearrangement translates into changes in the behavioral characteristics observed. We would suggest that changing the environment by introducing the element of movability of the altered objects is not independent of, but rather interacts with, other parameters of the environment, such as its complexity and spatial arrangement.

## Supporting information

S1 Data (CSV)
